# Prevalence of non-suicidal self-harm and service contact in England, 2000–14: repeated cross-sectional surveys of the general population

**DOI:** 10.1016/S2215-0366(19)30188-9

**Published:** 2019-07

**Authors:** Sally McManus, David Gunnell, Claudia Cooper, Paul E Bebbington, Louise M Howard, Traolach Brugha, Rachel Jenkins, Angela Hassiotis, Scott Weich, Louis Appleby

**Affiliations:** a National Centre for Social Research, London, UK; b Population Health Sciences, University of Bristol, Bristol UK; c National Institute of Health Research Biomedical Research Centre at the University Hospitals Bristol NHS Foundation Trust and the University of Bristol, Bristol, UK; d Division of Psychiatry, University College London, London, UK; e Camden and Islington NHS Foundation Trust, London, UK; f Section of Women's Mental Health, Institute of Psychiatry, Psychology and Neuroscience, King's College London, London, UK; g Health Services and Population Research Department, Institute of Psychiatry, Psychology and Neuroscience, King's College London, London, UK; h Department of Health Sciences, University of Leicester, Leicester, UK; i School of Health and Related Research, University of Sheffield, Sheffield, UK; j Division of Psychology and Mental Health, University of Manchester, Manchester, UK

## Abstract

**Background:**

The number of people presenting to hospital emergency departments after self-harming has increased in England. However, most people who self-harm do not present to hospitals, so whether this rise reflects an increase in the prevalence of self-harm in the community is unknown. Also unknown is whether the prevalence of non-suicidal self-harm (NSSH) or suicidal self-harm, or both, has increased. We aimed to establish temporal trends in the prevalence of NSSH in England.

**Methods:**

We analysed data from participants in the 2000 (n=7243), 2007 (n=6444), and 2014 (n=6477) Adult Psychiatric Morbidity Surveys of the general population, selecting those aged 16–74 years and living in England. We used weighted data and controlled for complex survey design. We generated temporal trends in lifetime prevalence and methods of, and motivations for, NSSH, and consequent service contact. We used multiple variable logistic regression analyses to investigate factors associated with service contact.

**Findings:**

The prevalence of self-reported lifetime NSSH increased from 2·4% (95% CI 2·0–2·8) in 2000, to 6·4% (5·8–7·2) in 2014. Increases in prevalence were noted in both sexes and across age groups—most notably in women and girls aged 16–24 years, in whom prevalence increased from 6·5% (4·2–10·0) in 2000, to 19·7% (15·7–24·5) in 2014. The proportion of the population reporting NSSH to relieve unpleasant feelings of anger, tension, anxiety, or depression increased from 1·4% (95% CI 1·0–2·0) to 4·0% (3·2–5·0) in men and boys, and from 2·1% (1·6–2·7) to 6·8% (6·0–7·8) in women and girls, between 2000 and 2014. In 2014, 59·4% (95% CI 54·7–63·9) of participants who had engaged in NSSH reported no consequent medical or psychological service contact, compared with 51·2% (42·2–60·0) in 2000 and 51·8% (47·3–56·4) in 2007. Male participants and those aged 16–34 years were less likely to have contact with health services than were female participants and older people.

**Interpretation:**

The prevalence of NSSH has increased in England, but resultant service contact remains low. In 2014, about one in five female 16–24-year-olds reported NSSH. There are potential lifelong implications of NSSH, such as an increased frequency of suicide, especially if the behaviours are adopted as a long-term coping strategy. Self-harm needs to be discussed with young people without normalising it. Young people should be offered help by primary care, educational, and other services to find safer ways to deal with emotional stress.

**Funding:**

NHS Digital, English Department of Health and Social Care, and the National Institute for Health Research.

## Introduction

The number of studies of non-suicidal self-harm (NSSH) or non-suicidal self-injury has grown worldwide since 2000,^1^ leading to improved recognition and informed prevention programmes.^2^ People who self-harm and present to hospital are at increased risk of suicide, fatal alcohol or drug poisoning, and other causes of mortality.^3^ Whether the prevalence or nature of NSSH is changing is unclear, because studies of temporal trends are rare and their findings inconsistent.^4^

A systematic review^5^ of NSSH in adolescent samples worldwide showed no evidence of an increased prevalence between 2005 and 2011. In most other studies, NSSH was amalgamated with suicide attempts.^6^, ^7^, ^8^, ^9^ Analyses of Danish hospital registration data for 1994–2011, for example, showed a rising frequency of self-harm (with and without suicidal intent) in both sexes, with the greatest increases in women and girls aged 15–24 years.^9^ Data from the National Self-Harm Registry in Ireland showed that the frequency of self-harm increased by 22% between 2007 and 2016 in 10–24-year-olds, and by more in women and girls.^8^ In Geulayov and colleagues' study^7^ of adults presenting to five general hospitals in England, the frequency of self-harm (with and without suicidal intent) fell between 2000 and 2012 in women and girls, and between 2000 and 2008 in men and boys, before increasing again. However, analysis of English primary care data showed that the incidence of self-harm (with and without suicidal intent) increased by 68% in girls aged 13–16 years between 2011 and 2014), but did not change in boys or in girls of other ages.^2^

**Research in context**

**Evidence before this study**

We searched PubMed with the terms “nonsuicidal self-harm” or “nonsuicidal self-injury” and “prevalence”, “rates”, and “trends” to identify articles published in English up to September, 2018. Available evidence for temporal trends in self-harm in England comes from studies of populations in contact with services. Findings are mixed, with some evidence that the prevalence of self-harm is increasing, particularly in young people. In most service use data, however, suicidal and non-suicidal self-harm (NSSH) are amalgamated, and temporal trends could reflect changes in patterns of help-seeking or treatment availability and coding. Findings based on people in contact with services might not be generalisable to the wider population, in whom much self-harm goes untreated.

**Added value of this study**

This study provides, to our knowledge, the first evidence of long-term trends in non-suicidal self-harm in the general population in England. We showed that prevalence of NSSH increased in both sexes and across all age groups between 2000 and 2014. This increase was mostly because of rises in self-cutting and increased use of NSSH to relieve unpleasant feelings. Absolute rises were highest in women and girls aged 16–24 years. Most participants reported no medical or psychological service contact after NSSH. Men and 16–34-year-olds were particularly unlikely to have service contact.

**Implications of all the available evidence**

The prevalence of NSSH has increased steeply. In 2014, one in five women and girls aged 16–24 years reported having self-harmed. NSSH in men could go unrecognised because they might be less likely both to seek and receive interventions. If self-harm is increasing among young people partly because it is thought of as a way of coping with emotional stress, there could be serious long-term public health implications, including normalisation of self-harm and potential increases in suicide rates. Better primary care and educational services need to be offered, and self-harm needs to be discussed in a way that helps young people to find safer ways of coping.

People presenting to hospitals or primary care differ in profile from the wider population engaging in self-harm: they are more likely to attempt suicide or overdose, but less likely to engage in NSSH or self-cutting.^10^ Furthermore, although many argue that the motives underpinning self-harm are multiple, fluid, and complex,^11^ effective intervention is likely to depend on understanding NSSH and suicide attempts as distinct issues.^12^ Additionally, much research done has focused on adolescents.^5^

We used a series of high-quality cross-sectional surveys of the general English population to establish trends in NSSH from 2000 to 2014. We examined changes in the prevalence of self-reported NSSH, the methods used, reported motivations, and reports of subsequent contact with medical or psychological services

## Methods

### Study design and participants

We did a secondary analysis of data from the 2000, 2007, and 2014 Adult Psychiatric Morbidity Surveys, a series of surveys of the mental health of the general population. Although the geographical and age range covered by the surveys varied (the 2000 wave included people aged 16–74 years living in England, Scotland, and Wales, whereas 2007 and 2014 waves covered England only and had no upper age limit to participate), for comparability we selected participants aged 16–74 years and living in England for our analysis (appendix p 1). Each wave of the Adult Psychiatric Morbidity Surveys had a similar stratified random probability sampling design suitable to produce a sample representative of the population living in private households in England. Sampling, procedure, and ethical review details have been previously published.^16^, ^17^, ^18^

Briefly, the first stage involved selection of addresses from the Postcode Address File,^13^ which covered 97% of households. People living in communal or institutional establishments or temporary housing, and homeless people were not sampled. Although these populations might have a higher prevalence of NSSH than the general population,^14^ they account for less than 2% of the overall population, and their exclusion should not affect overall estimates.^15^

Fieldwork took place from March to September, 2000; from October, 2006, to December, 2007; and from May, 2014, to September, 2015. Trained research interviewers visited addresses to identify private households with at least one resident aged 16 years or older. One person was randomly selected in each eligible household. Interviews were done in participants' own homes and took 1·5 h on average. Most of the questionnaire was administered face to face, but some sensitive information (eg, experience of abuse) was self-completed: participants keyed their responses into a laptop for enhanced privacy. Ethical approval was obtained from the relevant ethics committees. Our secondary analyses were approved by the National Centre for Social Research's ethical review committee.

### Measures

DSM-5^19^ includes non-suicidal self-injury and suicidal behaviour disorder as conditions for further study.^20^ Although intent can be difficult to establish,^21^ in the Adult Psychiatric Morbidity Surveys suicide attempts were distinguished from NSSH. In 2000, 2007, and 2014, participants were asked (face to face) “Have you ever deliberately harmed yourself in any way but not with the intention of killing yourself?” without further prompts. Affirmative responses to this question were used to establish lifetime NSSH. Our measure of NSSH is not equivalent to the DSM's non-suicidal self-injury because it does not take into account the frequency, recency, severity, or method of self-harm, or other criteria. Because our definition included self-injury and self-poisoning, we refer to NSSH rather than non-suicidal self-injury. Questions about NSSH were also included in the self-completion section in 2007 and 2014, but we used face-to-face responses to maximise comparability in trends with 2000. We also included a variable combining face-to-face and self-completed responses about NSSH, to provide the most recent and inclusive indication of prevalence. Only in 2014 was the most recent occasion of NSSH dated (which was coded as “past week”, “past year”, or “longer ago”). Suicide attempts were asked about with the question “Have you ever made an attempt to take your life, by taking an overdose of tablets or in some other way?”

Participants reporting NSSH were asked about methods, motivations, and subsequent service contact; the same wording was used in each wave of the Adult Psychiatric Morbidity Surveys. In 2000 and 2007, the follow-up questions were asked face to face, but in 2014 they were asked by self-completion. To ensure comparability between waves, we restricted trend measures for 2014 to participants who reported NSSH face to face. For the methods used, participants were asked “Did you cut yourself, or burn yourself, or swallow anything, or harm yourself some other way?” More than one method could be coded. Regarding motiviation, they were asked “Did you do any of these things to draw attention to your situation or to change your situation?” and “Did you do any of these things because it relieved unpleasant feelings of anger, tension, anxiety, or depression?” Participants could select neither, one, or both reasons. One variable was produced for whether participants reported being motivated by change, and one for whether participants reported being motivated by relieving unpleasant feelings (these categories were not mutually exclusive). For subsequent service contact, participants were asked “Have you received medical attention for deliberately harming yourself in any of these ways?” and “Have you ever seen a psychiatrist, psychologist, or counsellor because you had harmed yourself?” Participants were classified as having medical contact only, psychological contact only, both, or neither.

Because detailed classification of ethnicity varied somewhat between the waves, we used broad categories: white, black, Asian, and other or mixed. Housing was classified as owner-occupied or rented. People who had been in arrears with payments in the past year were identified—eg, disconnection from gas, electricity, or other fuel services because of failure to pay, being “seriously behind in paying within the time allowed” for a range of services and obligations. Highest educational qualifications and household income were elicited by showing participants lists of options, from which they could choose their response. We classified area-level deprivation into Index of Multiple Deprivation quintiles.

Symptoms of common mental disorders were measured with the Clinical Interview Schedule—Revised (CIS-R), an interviewer-administered structured interview that provides a continuous scale reflecting the overall severity of psychopathology in the week before interview.^22^ In our analyses, we divided participants' scores into two groups: 0–17 and 18 or higher. This threshold of 18 or higher was selected because it suggests a severity of symptoms for which intervention is warranted.^18^ Participants also self-rated their general health, and we grouped responses into two categories: excellent or very good, and good, fair, or poor.

### Statistical analysis

We used weighted data for our analyses, and took the complex design of the survey into account. Weighting adjusted for selection probabilities and non-response, thereby rendering results representative of the household population aged 16 years or older at the time of each survey. Population control totals were obtained from the UK Office for National Statistics mid-year population estimates for age by sex and region. Bases are presented unweighted. Non-overlapping 95% CIs provided statistical evidence for differences in prevalence between periods or subgroups. We did multiple variable logistic regression analyses of factors predicting service contact as a result of NSSH. We calculated unadjusted odds ratios (ORs) for service contact, and ran two models to calculate adjusted ORs. The first adjusted model included sex, age, tenure, debt arrears, CIS-R score, and general health. The second model additionally controlled for reported method of self-harm and whether the participant reported having ever made a suicide attempt. Missing data were excluded from analyses. All analyses were done in SPSS (version 21.0) or Stata (version 14.1).

### Role of the funding source

The funder had no role in the study design, data collection, data analysis, data interpretation, or writing of the report. The corresponding author had full access to all the data in the study and had final responsibility for the decision to submit for publication.

## Results

We analysed data for 7243 people aged 16–74 years in England in 2000, 6477 in 2007, and 6477 in 2014. Data for the number of households invited to participate, the number of people interviewed, and response rates are detailed in the appendix (p 1). Missing data were minimal (data not shown). Most missing data were a result of the 283 (4·3%) participants in the 2014 survey who did not provide responses for the self-completion section (data not shown). These participants were significantly older (p<0·0001) and had higher scores on the Clinical Interview Schedule—Revised (p=0·0010) than those who completed the self-completion section (appendix p 2). Self-completion non-response was not associated with sex (appendix p 2).

Overall, the lifetime prevalence of NSSH rose from 2·4 (95% CI 2·0–2·8) in 2000, to 3·8 (3·3–4·3) in 2007, and to 6·4 (5·8 to 7·2) in 2014. Table 1 shows the demographic characteristics of the analysed sample weighted to the age–sex–region profile of the wider population at the time of each survey. Across the three waves of the Adult Psychiatric Morbidity Surveys, the profile remained stable in terms of age and sex, but the proportion of respondents who were white fell (from 92·6% in 2000 to 89·4% in 2007 and 86·3% in 2014; table 1). In each wave, NSSH was most prevalent in the youngest age groups and least prevalent in the oldest age groups (table 1). The prevalence of NSSH did not differ significantly between ethnic groups in any wave (table 1). Prevalence was similar in male and female participants in 2000 and 2007, but was significantly higher in women and girls (7·9% [95% CI 6·9–9·0]) than in men and boys (5·0% [4·0–6·1]) in 2014 (p=0·0002; table 1). Prevalence increased in both sexes and in all age groups (table 1).

**Table 1:** Prevalence of non-suicidal self-harm ever (face-to-face report) in 16–74-year-olds in England in 2000, 2007, and 2014

	**2000 (n=7243)**			**2007 (n=6444)**			**2014 (n=6477)**		
	n (%)*	Reported non-suicidal self-harm (% [95% CI])	p value	n (%)*	Reported non-suicidal self-harm (% [95% CI])	p value	n (%)*	Reported non-suicidal self-harm (% [95% CI])	p value
**Sex**									
Male	3237 (49·9%)	65 (2·1% [1·6–2·7])	0·136	2824 (49·4%)	98 (3·7% [3·0–4·5])	0·759	2638 (49·6%)	119 (5·0% [4·0–6·1])	0·0002
Female	4006 (50·1%)	105 (2·7% [2·2–3·4])	..	3620 (50·6%)	131 (3·8% [3·1–4·7])	..	3839 (50·4%)	291 (7·9% [6·9–9·0])	..
**Age, years**									
16–24	665 (14·7%)	37 (5·3% [3·7–7·6])	<0·0001	567 (15·5%)	59 (8·9% [6·9–11·6])	<0·0001	559 (15·7%)	90 (13·7% [11·2–16·7])	<0·0001
25–34	1441 (20·6%)	60 (3·8% [2·8–5·0])	..	1035 (18·1%)	58 (4·6% [3·5–6·1])	..	1034 (18·7%)	117 (10·3% [8·4–12·6])	..
35–44	1540 (20·8%)	42 (2·5% [1·8–3·4])	..	1409 (21·1%)	67 (4·7% [3·6–6·1])	..	1178 (17·9%)	87 (6·4% [5·1–8·0])	..
45–54	1331 (18·5%)	18 (1·0% [0·6–1·7])	..	1128 (17·7%)	30 (2·1% [1·4–3·1])	..	1293 (19·3%)	55 (2·9% [2·2–3·9])	..
55–64	1194 (14·1%)	11 (0·9% [0·5–1·6])	..	1278 (16·3%)	14 (0·9% [0·5–1·6])	..	1226 (15·4%)	45 (3·3% [2·4–4·6])	..
65–74	1071 (11·4%)	2 (0·1% [0·0–0·5])	..	1027 (11·3%)	1 (0·1% [0·0–0·8])	..	1187 (13·0%)	16 (1·1% [0·7–2·0])	..
**Ethnicity**†									
White	6739 (92·6%)	159 (2·5% [2·1–2·9])	0·706	5876 (89·4%)	211 (3·9% [3·3–4·5])	0·239	5779 (86·3%)	370 (6·5% [5·8–7·3])	0·743
Black	179 (2·5%)	5 (1·6% [0·6–3·8])	..	183 (3·3%)	4 (3·2% [1·1–9·2])	..	182 (3·2%)	10 (6·6% [2·9–13·9])	..
Asian	151 (3·0%)	3 (1·6% [0·5–5·0])	..	196 (4·2%)	4 (1·0% [0·3–3·8])	..	345 (7·6%)	18 (5·8% [3·4–9·7])	..
Other or mixed	127 (1·9%)	2 (1·7% [0·4–6·7])	..	156 (3·2%)	8 (4·5% [2·2–9·2])	..	147 (2·8%)	9 (4·6% [2·2–9·1])	..

The table shows data for all participants with valid data for non-suicidal self-harm (appendix p 1).

* All percentages are presented weighted and bases unweighted.

† Ethnicity data were missing for 47 people in 2000, 33 people in 2007, and 24 people (three of whom had reported non-suicidal self-harm) in 2014.

**Figure 1:**
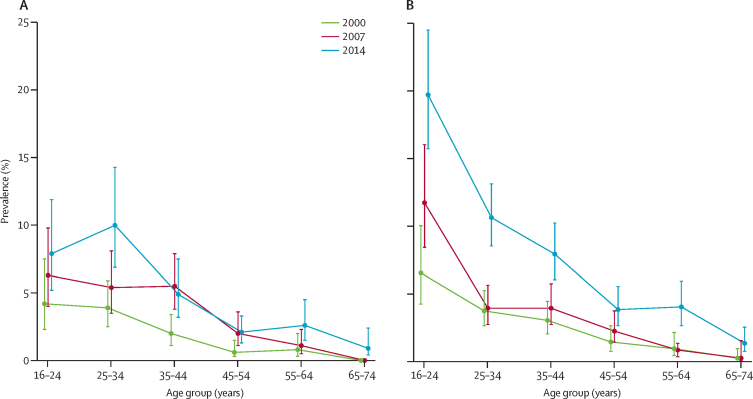
Prevalence of non-suicidal self-harm in men and boys (A) and women and girls (B), by age group Data were self-reported in person (ie, face to face). Error bars show the 95% CIs.

Prevalence increased in several age-by-sex groups; the percentage point increase in absolute terms was greatest in girls and young women (figure 1). In 2014 19·7% (95% CI 15·7–24·5) of female 16–24-year-olds reported NSSH in face-to-face interviews, compared with 6·5% (4·2–10·0) in 2000, and 11·7% (8·4–16·0) in 2007 (appendix p 3). Self-completion reports of NSSH were available for 2014, and suggest underreporting in the face-to-face reports. When self-completion reports were included, 25·7% (95% CI 21·0–31·0) of women and girls aged 16–24 years reported NSSH (appendix p 3).

**Figure 2:**
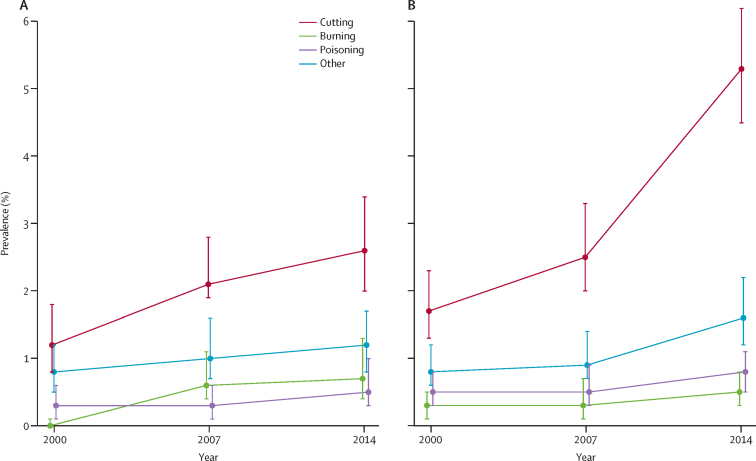
Method of non-suicidal self-harm reported by men and boys (A) and women and girls (B) aged 16–74 years Error bars show the 95% CIs.

In each survey wave, around two-thirds of participants reporting NSSH had self-cut (appendix p 4). As NSSH became more common, the overall population prevalence of self-cutting also increased, from 1·5% (95% CI 1·2–1·8) in 2000, to 3·9% (3·5–4·5) in 2014 (appendix p 4). The increase was particularly pronounced in women and girls, in whom prevalence rose from 1·7% (1·3–2·3) in 2000, to 5·3% (4·5–6·2) in 2014, with the bulk of the increase occurring since 2007 (figure 2). In 2000 (p=0·129) and 2007 (p=0·334), the prevalence of self-cutting did not differ significantly by sex, whereas in 2014 it was higher in women and girls than in men and boys (p<0·0001; appendix p 4). In men and boys, the prevalence of self-burning increasing from 0·0% (95% CI 0·0–0·1) in 2000, to 0·6% (0·4–1·1) in 2007, and 0·7% (0·4–1·3) in 2014 (figure 2). The prevalence of self-poisoning remained stable in both sexes (figure 2). When participants in 2014 who reported NSSH in the self-completion section of the interview were also included, 7·0% (95% CI 6·1–8·0) of women and girls and 3·4% (2·7–4·3) of men and boys reported self-cutting (appendix p 4).

**Figure 3:**
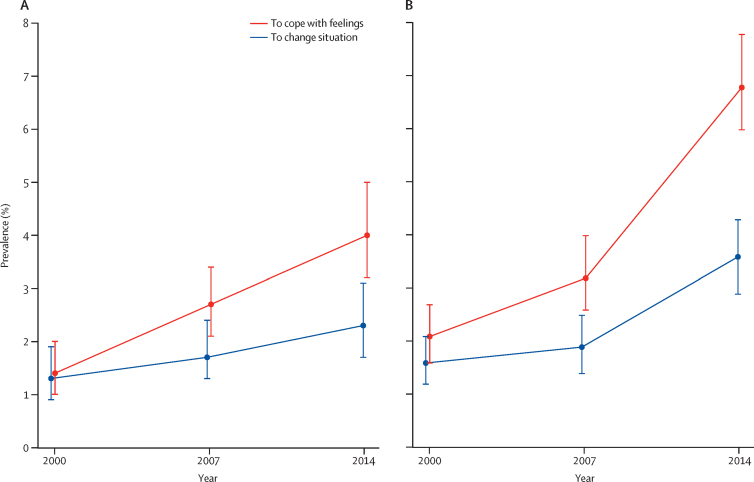
Reasons for non-suicidal self-harm among men and boys (A) and women and girls (B) aged 16–74 years Error bars show the 95% CIs.

The proportion of the population reporting NSSH to relieve unpleasant feelings of anger, tension, anxiety, or depression roughly tripled in prevalence in both sexes between 2000 and 2014, from 1·4% (95% CI 1·0–2·0) to 4·0% (3·2–5·0) in men and boys, and from 2·1% (1·6–2·7) to 6·8% (6·0–7·8) in women and girls (figure 3). In 2000 and 2007, the prevalence of NSSH to cope with these feelings did not differ significantly by sex, but it was significantly more common in women and girls than in men and boys in 2014 (figure 3; appendix p 5). The prevalence of NSSH to cope with feelings was highest in women and girls aged 16–24 (17·7% [95% CI 13·9–22·3]). The corresponding prevalence in male 16–24-year-olds was 5·8% (95% CI 3·6–9·3). When those who reported NSSH in the self-completion section in 2014 were included, 22·4% (95% CI 18·0–27·5) of female 16–24-year-olds reported NSSH to cope (appendix p 5). Overall, 14·5% (95% CI 11·8–17·7) of people aged 16–24 years reported using NSSH to cope with feelings (appendix p 5). The proportion of participants reporting NSSH to change their situation increased overall, but less steeply than the proportion using NSSH as a coping mechanism (figure 3). The proportion of participants using NSSH to change their situation did not differ by sex in any of the three waves (figure 3; appendix p 5).

The proportion of people who engaged in NSSH and reported no subsequent medical or psychological service contact remained stable between 2000 (51·2% [95% CI 42·2–60·0]) and 2007 (51·8% [47·3–56·4]), but increased somewhat in 2014 (59·4% [54·7–63·9]), although this increase was not significant (table 2). When participants in 2014 who reported NSSH only in the self-completion section were included, 62·6% (95% CI 58·9–66·1) reported no medical or psychological service contact after NSSH (table 2).

**Table 2:** Medical or psychological service contact as a result of non-suicidal self-harm in 16–74-year-olds in England, 2000, 2007, and 2014

	**n (% [95% CI])**
**2000**	
No medical or psychological contact	87 (51·2% [42·2–60·0])
Medical contact only	22 (14·6% [9·2–22·4])
Psychological contact only	22 (13·2% [8·2–20·8])
Both medical and psychological contact	39 (20·9% [14·9–28·5])
**2007**	
No medical or psychological contact	118 (51·8% [47·3–56·4])
Medical contact only	16 (7·1% [5·1–9·8])
Psychological contact only	41 (18·3% [14·4–23·1])
Both medical and psychological contact	54 (22·7% [18·8–27·1])
**2014**	
No medical or psychological contact	169 (59·4% [54·7–63·9])
Medical contact only	18 (4·0% [2·8–5·7])
Psychological contact only	54 (15·5% [13·1–18·3])
Both medical and psychological contact	77 (21·1% [17·7–25·0])
**2014 (including self-completion reporting)**	
No medical or psychological contact	242 (62·6% [58·9–66·1])
Medical contact only	28 (4·6% [3·5–6·0])
Psychological contact only	59 (13·0% [11·1–15·2])
Both medical and psychological contact	95 (19·9% [17·0–23·1])

Women and girls who engaged in NSSH had roughly twice the odds of medical or psychological service contact that men and boys had (unadjusted OR 1·99 [95% CI 1·22–3·25]; table 3). Adjustment for sex, age, tenure, debt arrears, mental health, general health, method of self-harm, and ever attempting suicide, did not reduce these odds (table 3). Service contact after NSSH was also higher in people aged 35–74 years than in those aged 16–34 years in both adjusted and unadjusted analyses (table 3). People living in rented accommodation were more likely than owner-occupiers to have contact with services in all three analyses (table 3). None of the other socioeconomic indicators tested, such as educational qualifications, household income, and area-level deprivation, was significant, and they were therefore not retained in models (data not shown).

**Table 3:** Odds ratios for self-reported medical or psychological service contact as a result of non-suicidal self-harm in 16–74-year-olds in England, 2014

		**Unadjusted**		Model 1*		**Model 2**†	
		Odds ratio (95% CI)	p value	Odds ratio (95% CI)	p value	Odds ratio (95% CI)	p value
**Sex**							
	Male	Reference	..	Reference	..	Reference	..
	Female	1·99 (1·22–3·25)	0·006	2·40 (1·41–4·08)	0·001	2·49 (1·43–4·25)	0·001
**Age, years**							
	16–34	Reference	..	Reference	..	Reference	..
	35–74	1·67 (1·08–2·57)	0·022	1·89 (1·15–3·10)	0·012	1·82 (1·06–3·11)	0·029
**Tenure**							
	Owner occupier	Reference	..	Reference	..	Reference	..
	Renter	2·65 (1·64–4·29)	<0·0001	2·58 (1·47–4·51)	0·001	2·59 (1·45–4·63)	0·001
**In debt arrears**							
	Not in arrears	Reference	..	Reference	..	Reference	..
	Arrears in past year	1·16 (0·68–1·96)	0·587	0·76 (0·42–1·39)	0·378	0·67 (0·36–1·24)	0·202
**CIS–R score**							
	CIS–R less than 18	Reference	..	Reference	..	Reference	..
	CIS–R 18 or more	2·43 (1·51–3·91)	<0·0001	1·95 (1·15–3·31)	0·013	1·42 (0·80–2·52)	0·234
**General health**							
	Excellent or very good	Reference	..	Reference	..	Reference	..
	Poor, fair or good	2·78 (1·74–4·45)	<0·0001	2·00 (1·17–3·42)	0·011	1·63 (0·93–2·87)	0·090
Self-harm method							
	Used other method only	Reference	..	..	..	Reference	..
	Self-cut	1·37 (0·87–2·15)	0·170	..	..	1·31 (0·77–2·22)	0·314
Ever attempted suicide							
	Had not made an attempt	Reference	..	..	..	Reference	..
	Had made a suicide attempt	4·13 (2·58–6·62)	<0·0001	..	..	3·25 (1·91–5·53)	<0·0001

CIS-R=Clinical Interview Schedule—Revised.

* Adjusted for sex, age, tenure, debt arrears, mental health, and general health.

† Adjusted for sex, age, tenure, debt arrears, mental health, general health, method of self-harm, and ever attempting suicide.

People with worse mental (unadjusted OR 2·43 [95% CI 1·51–3·91]) or general (2·78 [1·74–4·45]) health were more likely to have contact with services after NSSH than were those with better mental or general health. After adjustment for the nature of self-harming behaviour, these associations weakened, and were no longer significant (table 3). In both unadjusted (OR 4·13 [95% CI 2·58–6·62]) and adjusted (3·25 [1·91–5·53]) analyses, the odds of reporting service contact after NSSH were increased in people who had at some point also made a suicide attempt (table 3).

## Discussion

In an analysis of data from high-quality cross-sectional surveys of the English population, we noted steep increases in the lifetime prevalence of self-reported NSSH between 2000 and 2014. This increase was evident in both men and boys and in women and girls, and across all age groups. The absolute rise was greatest in female 16–24-year-olds, in whom the proportion increased from 6·5% (95% CI 4·2–10·0) in 2000, to 19·7% (15·7–24·5) in 2014 (with the bulk of the increase occurring since 2007).

Sex specific rises in self-harm (with and without suicidal intent) have previously been reported in service settings in Denmark,^9^ Ireland,^8^ and England.^2^ The rise in the prevalence of NSSH in our study was largely because of an increased prevalence of self-cutting, from 1·5% (95% CI 1·2–1·8) in 2000, to 3·9% in 2014 (3·5–4·5). This increase was more pronounced in women and girls than in men and boys, and in 2014 self-cutting was significantly more common in female than in male participants. We found no evidence of an increase in self-poisoning. There was some indication of an increase in self-burning among men between 2000 and 2007, although numbers were low and this finding should be treated with caution. Our findings for self-cutting and self-poisoning were consistent with those of previous research.^7^

The number of people using NSSH to relieve unpleasant feelings of anger, tension, anxiety, or depression roughly tripled between 2000 and 2014, and the prevalence of NSSH to try to change a situation roughly doubled. Although the use of NSSH as a coping strategy increased steeply across the population, it was most pronounced in young people. More than 10% of young people reported having self-harmed to relieve unpleasant feelings of anger, tension, anxiety, or depression in the 2014 wave. This finding is important because individuals who start to self-harm when young might adopt the behaviour as a long-term coping strategy.^12^ There is also a risk that the behaviour could lead in time to increases in suicides and suicide attempts.^30^ Since 2010, an upward trend in suicide has been evident among people aged younger than 20 years in the UK.^29^ Anxiety and depression in the English general population have also increased in children^31^ and young women.^18^ The factors underlying these adverse trends in young people's mental health are unclear and require further research to inform appropriate prevention strategies.^32^

At least half of those who reported NSSH also reported no subsequent medical or psychological contact as a result, consistent with the findings of previous research.^10^ Thus, studies of the prevalence of NSSH in service users will be affected by the determinants of contact. In 2014, in our analyses, contact with health services was less likely in male participants than in female participants, and in younger participants than in older participants (in analyses adjusted for sex, age, tenure, debt arrears, mental health, general health, method of NSSH and previous suicide attempts). Service contact after NSSH was more common among people with poor general or mental health than among those with good health, perhaps because they were more likely to be in contact with services already. We also found that service contact was increased in those who had also made a suicide attempt, perhaps because of a need for medical treatment. Overall, between 2000 and 2014, we noted no evidence of an increase in treatment contact among people who self-harmed, suggesting that changes in the prevalence of NSSH in health-care settings probably reflect changes in the community prevalence of NSSH.

The repeat probability-sample surveys of whole adult populations with consistent methods that we analysed provide valuable evidence about temporal trends in NSSH. However, such surveys inevitably have limitations. First, precision of some estimates is low because of the small numbers of participants reporting NSSH, especially in the 2000 survey, in which the prevalence of NSSH was lowest. Second, the findings could be subject to bias due to non-participation, although the response rate—69% in 2000, and 57% in 2007 and 2014—was in line with that in similar surveys.^23^ Mental health is associated with the propensity to take part in surveys and could affect our estimates of the prevalence of NSSH.^24^ The development of non-response weights to address participation biases from known characteristics have been described previously.^18^ However, non-response weighting has little effect on results, suggesting that non-responders were similar to responders in many characteristics. Third, in terms of balancing comparability and validity, we prioritised consistency over improvement or updating of survey questions. The issue of self-harm intent is complex and the answers that participants could select about motivations were reductive. Even with open questions, reasons given could reflect subsequent rationalisations.^21^ Use of the word “attention” could have been interpreted as an implication that NSSH is attention seeking, which could have led to people not choosing endorsing this option. The coping or affect regulation model of self-harm is now much more widely accepted.^25^ Although methods largely remained consistent across the three survey waves, mode changes could have affected the trends reported. In 2000 and 2007, the follow-up questions were asked face to face, whereas in 2014 they were part of the self-completion interview. More socially stigmatised feelings and behaviours might thus be underreported in 2000 and 2007.^26^ To maximise cross-wave comparability, data for methods of, reasons for, and service contact after NSSH were restricted to those participants who reported NSHH face to face. These prevalences should thus be considered consistent underestimates.

Fourth, some of the increased reporting of NSSH could reflect changing conceptualisations of NSSH: behaviours that people did not deem NSSH in previous surveys could be more likely to be included in 2014. Furthermore, as NSSH has become less stigmatised, some people might have felt more able to disclose it in later surveys.^27^ Previous underreporting could mean that the actual increase in NSSH is less pronounced than our findings suggest. Fifth, the sample was too small for robust analysis by ethnic group, and questions might not have captured how NSSH manifests in different ethnic groups. No option was presented for punching or hitting against something—methods of NSSH that are more common in men than in women.^28^ Although these methods of NSSH could have been captured by the “other” response option, not having a direct prompt could have led to underreporting. Finally, our lifetime indicator did not take into account frequency or recency of NSSH. Although participants were asked about engaging in NSSH in the past year in the 2014 survey, this question was not included in 2000 or 2007, so trends could not be examined.

In conclusion, we found an increase in the prevalence of NSSH in all age groups in England, but particularly in young women and girls. An increase in the prevalence of using self-harm to cope with emotional stress could have serious long-term public health implications. There is a risk that self-harm will become normalised for young people. Furthermore, NSSH increases the risk of later suicide; a cohort effect is possible by which suicide rates in these groups could potentially increase. Young people need health and educational services to be available, and health and other professionals need to discuss self-harm with young people and encourage them to find safer ways of coping.

## Data sharing

The Adult Psychiatric Morbidity Surveys datasets are in the UK Data Service archive. NHS Digital manages the survey series and reviews requests for access to the latest dataset. Requests for access to the 2014 dataset that we used in this analysis should be made to the Data Access Request Service at NHS Digital.
