# Supplementary material for: Prevalence of non-suicidal self-harm and service contact in England, 2000–14: repeated cross-sectional surveys of the general population
Source: Lancet Psychiatry. 2019 Jul;6(7):573–81. doi: 10.1016/S2215-0366(19)30188-9 (PMC7646286; doi:10.1016/S2215-0366(19)30188-9)
Supplement: Supplementary appendix [file mmc1.pdf]

THE LANCET
Psychiatry

Supplementary appendix

This appendix formed part of the original submission and has been peer reviewed. We post it as supplied by the authors.

Supplement to: McManus S, Gunnell D, Cooper C, et al. Prevalence of non-suicidal self-harm and service contact in England, 2000–14: repeated cross-sectional surveys of the general population. *Lancet Psychiatry* 2019; published online June 4. http://dx.doi.org/10.1016/S2215-0366(19)30188-9.

Prevalence of non-suicidal self-harm and service contact in England, 2000-14: Supplementary materials

Supplementary Table 1. APMS response rate

	**2000[a]**	**2007[b]**	**2014[b]**
Target households	12792	13171	13313
Interviewed adults	8886	7461	7546
Response rate	69%	57%	57%
Living in England	7247	7461	7546
And aged 16-74	7247	6453	6484
And with valid data on NSSH	7243	6444	6477

[a] APMS 2000 covered 16 to 74 year olds living in England, Scotland, or Wales.
[b] APMS 2007 and 2014 covered those aged 16 years or over living in England.

Supplementary Table 2. Factors associated with doing the self-completion part of the interview in APMS 2014[a]

		Adjusted Odds Ratio	P value	95% CI	
				Upper	Lower
**Sex**	Men	Ref			
	Women	0.99	0.939	0.76	1.29
**Age**	16 to 24	Ref	<0.0001		
	25 to 34	0.27	0.002	0.12	0.63
	35 to 44	0.26	<0.0001	0.12	0.53
	45 to 54	0.31	0.002	0.15	0.65
	55 to 64	0.18	<0.0001	0.09	0.37
	65 to 74	0.16	<0.0001	0.07	0.34
**Ethnic group**	White	Ref	<0.0001		
	Black/Black British	0.65	0.200	0.34	1.25
	Asian/Asian British	0.45	0.002	0.27	0.74
	Mixed, multiple, other	0.23	<0.0001	0.12	0.45
**Clinical Interview Schedule – revised (CIS-R)[b] score**	0 to 5	Ref	0.0010		
	6 to 11	0.81	0.258	0.57	1.16
	12 to 17	0.90	0.661	0.57	1.44
	18 or more	0.48	<0.0001	0.32	0.71

[a] In the 2014 survey 283 participants (4.8%) aged 16 to 74 choose to not do the self-completion. These participations were more likely to be aged at least 25, to be Asian or of mixed or other ethnicity, and to have severe symptoms of common mental disorder (CIS-R 18+) than those who did do the self-completion.
[b] Higher CIS-R score indicates greater severity of symptoms of common mental disorder.

Supplementary Table 3. Non-suicidal self-harm ever (reported face to face) by age and sex in England, 2000, 2007 and 2014

	16-24		25-34		35-44		45-54		55-64		65-74		16-74	
	%	95% CI	%	95% CI	%	95% CI	%	95% CI	%	95% CI	%	95% CI	%	95% CI
**2000**														
Men	4·2	2·3-7·5	3·9	2·5-5·9	2·0	1·1-3·4	0·6	0·3-1·5	0·8	0·3-2·0	-		2·1	1·6-2·7
Women	6·5	4·2-10·0	3·7	2·6-5·2	3·0	2·0-4·4	1·4	0·7-2·6	0·9	0·4-2·1	0·2	0·1-0·9	2·7	2·2-3·4
**2007**														
Men	6·3	4·0-9·8	5·4	3·5-8·1	5·5	3·8-7·9	2·0	1·1-3·6	1·1	0·5-2·3	-		3·7	3·0-4·5
Women	11·7	8·4-16·0	3·9	2·7-5·6	3·9	2·7-5·7	2·2	1·4-3·7	0·8	0·3-1·3	0·2	0·0-1·5	3·8	3·1-4·7
**2014**														
Men	7·9	5·2-11·9	10·0	6·9-14·3	4·9	3·2-7·5	2·1	1·3-3·3	2·6	1·5-4·5	0·9	0·4-2·4	5·0	4·0-6·1
Women	19·7	15·7-24·5	10·6	8·5-13·1	7·9	6·0-10·2	3·8	2·6-5·5	4·0	2·6-5·9	1·3	0·7-2·5	7·9	6·9-9·0
**Non-suicidal self-harm ever (reported face to face or by self-completion) by age and sex in England, 2014**														
Men	9·7	6·7-13·8	10·9	7·7-15·3	6·6	4·7-9·3	3·3	2·1-5·0	3·3	1·9-5·6	2·0	1·1-3·9	6·2	5·2-7·3
Women	25·7	21·0-31·0	13·2	10·9-16·0	9·2	7·2-11·8	5·0	3·6-6·7	5·0	3·4-7·2	1·8	1·0-3·2	10·0	8·9-11·2
All	17·5	14·6-20·8	12·1	10·0-14·5	7·9	6·5-9·6	4·1	3·2-5·3	4·1	3·0-5·6	1·9	1·3-3·0	8·1	7·4-8·9
**Non-suicidal self-harm in past year (reported face to face or by self-completion) by age and sex in England, 2014**														
Men	4.1	2.4-7.0	1.9	0.8-4.4	1.1	0.4-2.8	1.4	0.7-2.6	0.1	0.0-0.9	0.4	0.1-1.4	1.5	1.1-2.2
Women	9.1	6.3-12.9	2.3	1.4-3.7	1.7	1.0-2.9	0.7	0.4-1.4	0.8	0.4-1.8	0.1	0.0-0.6	2.4	1.9-3.1
All	6.5	4.8-8.8	2.1	1.3-3.2	1.4	0.8-2.3	1.1	0.6-1.7	0.5	0.2-1.0	0.2	0.1-0.7	2.0	1.6-2.4

Supplementary Table 4. Methods of non-suicidal self-harm ever used among 16-74-year olds, by sex in 2000, 2007 and 2014

	**Men**		**Women**		**All**		**p-value**
	%	95% CI	%	95% CI	%	95% CI	for assoc. with sex
**2000**	n=3237		n=4006		n=7243		
Cutting	1·2	0·8-1·8	1·7	1·3-2·3	1·5	1·2-1·8	0·129
Burning	0·0	0·0-0·1	0·3	0·1-0·5	0·1	0·1-0·3	0·013
Poisoning	0·3	0·1-0·6	0·5	0·3-0·8	0·4	0·2-0·6	0·321
Other	0·8	0·5-1·2	0·8	0·6-1·2	0·8	0·6-1·1	0·839
**2007**	n=2824		n=3620		n=6444		
Cutting	2·1	1·9-2·8	2·5	2·0-3·3	2·3	1·9-2·8	0·334
Burning	0·6	0·4-1·1	0·3	0·1-0·7	0·5	0·3-0·7	0·179
Poisoning	0·3	0·1-0·6	0·5	0·3-0·9	0·4	0·3-0·6	0·187
Other	1·0	0·7-1·6	0·9	0·7-1·4	1·0	0·8-1·3	0·706
**2014**	n=2638		n=3839		n=6477		
Cutting	2·6	2·0-3·4	5·3	4·5-6·2	3·9	3·5-4·5	<0·001
Burning	0·7	0·4-1·3	0·5	0·3-0·8	0·6	0·4-0·9	0·268
Poisoning	0·5	0·3-1·0	0·8	0·5-1·1	0·7	0·5-0·9	0·283
Other	1·2	0·8-1·7	1·6	1·2-2·2	1·4	1·1-1·8	0·199

2014, including those only reporting NSSH by self-completion

**2014**	n=2501		n=3646		n=6147		
Cutting	3·4	2·7-4·3	7·0	6·1-8·0	5·2	4·6-5·8	<0·001
Burning	0·9	0·5-1·4	0·6	0·4-0·9	0·7	0·5-1·0	0·280
Poisoning	0·9	0·5-1·4	1·1	0·8-1·5	1·0	0·7-1·3	0·534
Other	1·6	1·2-2·3	2·5	1·9-3·2	2·1	1·7-2·5	0·066

Supplementary Table 5. Self-reported motivations for non-suicidal self-harm in 16-74-year-olds, 2000, 2007 and 2014

Engaged in NSSH in order to cope with unpleasant feelings

	16-24		25-34		35-44		45-54		55-64		65-74		16-74	
	%	95% CI	%	95% CI	%	95% CI	%	95% CI	%	95% CI	%	95% CI	%	95% CI
**2000**														
Men	3·4	1·7-6·7	2·1	1·2-3·7	1·7	0·9-3·1	0·3	0·1-0·9	0·4	0·1-1·4	-	-	1·4	1·0-2·0
Women	5·8	3·6-9·1	2·9	2·0-4·4	2·3	1·4-3·6	0·5	0·2-1·1	0·6	0·2-1·6	0·2	0·1-0·9	2·1	1·6-2·7
All	4·6	3·1-6·7	2·5	1·8-3·5	2·0	1·4-2·9	0·4	0·2-0·8	0·5	0·2-1·1	0·1	0·0-0·5	1·8	1·4-2·2
**2007**														
Men	4·3	2·0-7·5	3·3	2·0-5·6	4·4	3·0-6·6	1·7	0·9-3·3	0·7	0·3-1·8	-	-	2·7	2·1-3·4
Women	9·4	6·6-13·3	3·7	2·5-5·3	3·3	2·2-5·1	1·8	1·0-3·2	0·7	0·3-1·7	0·2	0·0-1·5	3·2	2·6-4·0
All	6·9	5·1-9·2	3·5	2·5-4·8	3·9	2·9-5·2	1·7	1·1-2·7	0·7	0·4-1·3	0·1	0·0-0·8	3·0	2·5-3·5
**2014**														
Men	5·8	3·6-9·3	8·8	5·9-13·1	3·6	2·2-5·9	1·8	1·0-3·0	2·0	1·1-3·8	0·7	0·2-1·8	4·0	3·2-5·0
Women	17·7	13·9-22·3	9·4	7·4-11·8	6·9	5·1-9·1	3·1	2·0-4·6	3·1	2·0-4·8	0·6	0·2-1·5	6·8	6·0-7·8
All	11·6	9·3-14·5	9·1	7·3-11·4	5·3	4·1-6·7	2·4	1·7-3·4	2·6	1·8-3·7	0·6	0·3-1·3	5·4	4·8-6·1
**2014, including those only reporting NSSH by self-completion**														
Men	7·0	4·6-10·6	9·3	6·3-13·5	4·8	3·2-7·2	1·8	1·0-3·0	2·1	1·1-3·9	1·6	0·8-3·3	4·6	3·8-5·6
Women	22·4	18·0-27·5	11·6	9·4-14·3	7·8	5·9-10·3	4·1	2·9-5·7	3·8	2·5-5·6	0·9	0·4-2·1	8·5	7·5-9·5
All	14·5	11·8-17·7	10·4	8·5-12·8	6·3	5·1-7·9	2·9	2·2-3·9	2·9	2·1-4·1	1·3	0·7-2·2	6·5	5·9-7·3

Engaged in NSSH in order to try to change situation

	16-24		25-34		35-44		45-54		55-64		65-74		16-74	
	%	95% CI	%	95% CI	%	95% CI	%	95% CI	%	95% CI	%	95% CI	%	95% CI
**2000**														
Men	2·6	1·2-5·9	2·6	1·5-4·5	0·9	0·4-2·2	0·5	0·2-1·4	0·8	0·3-2·0	-	-	1·3	0·9-1·9
Women	4·2	2·4-7·2	1·7	1·0-2·9	1·8	1·1-3·1	0·9	0·5-1·8	0·5	0·2-1·3	-	-	1·6	1·2-2·1
All	3·4	2·2-5·4	2·2	1·4-3·2	1·4	0·9-2·1	0·7	0·4-1·3	0·7	0·3-1·3	-	-	1·4	1·2-1·8
**2007**														
Men	3·1	1·6-6·0	2·3	1·2-4·6	2·3	1·4-3·9	1·3	0·7-2·7	0·6	0·2-1·5	-	-	1·7	1·3-2·4
Women	5·0	3·0-8·2	2·4	1·5-3·8	2·2	1·2-3·8	0·9	0·4-2·0	0·4	0·1-1·3	0·2	0·0-1·5	1·9	1·4-2·5
All	4·0	2·7-6·0	2·4	1·6-3·6	2·2	1·5-3·3	1·1	0·6-1·9	0·5	0·2-1·0	0·1	0·0-0·8	1·8	1·5-2·2
**2014**														
Men	3·4	1·6-7·2	4·9	2·9-8·2	1·9	1·0-3·4	0·7	0·3-1·7	1·8	0·9-3·6	0·4	0·1-1·2	2·3	1·7-3·1
Women	7·3	4·8-10·9	4·8	3·3-6·8	3·5	2·4-5·2	2·1	1·4-3·3	2·8	1·7-4·5	0·6	0·3-1·4	3·6	2·9-4·3
All	5·3	3·7-7·7	4·8	3·5-6·6	2·7	1·9-3·8	1·4	1·0-2·1	2·3	1·5-3·4	0·5	0·2-1·1	2·9	2·5-3·5
**2014, including those only reporting NSSH by self-completion**														
Men	3·4	1·6-7·2	5·0	3·0-8·3	2·1	1·2-3·7	1·5	0·7-3·1	1·8	0·9-3·6	0·8	0·3-1·9	2·5	1·9-3·4
Women	9·2	6·3-13·1	5·3	3·7-7·4	4·5	3·1-6·5	2·9	2·0-4·2	3·6	2·3-5·6	0·9	0·4-1·8	4·4	3·7-5·2
All	6·2	4·4-8·7	5·2	3·8-6·9	3·3	2·4-4·5	2·2	1·5-3·1	2·7	1·9-3·9	0·8	0·5-1·5	3·5	3·0-4·1
